# Aryl hydrocarbon receptor (AHR) is a potential tumour suppressor in pituitary adenomas

**DOI:** 10.1530/ERC-17-0112

**Published:** 2017-06-22

**Authors:** R Formosa, J Borg, J Vassallo

**Affiliations:** 1 Department of MedicineFaculty of Medicine and Surgery, University of Malta, Msida, Malta; 2 Department of Applied Biomedical ScienceFaculty of Health Sciences, University of Malta, Msida, Malta; 3 Department of MedicineNeuroendocrine Clinic, Mater Dei Hospital, Msida, Malta

**Keywords:** AHR, tumour suppressor, microarray, pituitary adenoma

## Abstract

Pituitary adenomas (PA) represent the largest group of intracranial neoplasms and yet the molecular mechanisms driving this disease remain largely unknown. The aim of this study was to use a high-throughput screening method to identify molecular pathways that may be playing a significant and consistent role in PA. RNA profiling using microarrays on eight local PAs identified the aryl hydrocarbon receptor (AHR) signalling pathway as a key canonical pathway downregulated in all PA types. This was confirmed by real-time PCR in 31 tumours. The AHR has been shown to regulate cell cycle progression in various cell types; however, its role in pituitary tissue has never been investigated. In order to validate the role of AHR in PA behaviour, further functional studies were undertaken. Over-expression of AHR in GH3 cells revealed a tumour suppressor potential independent of exogenous ligand activation by benzo α-pyrene (BαP). Cell cycle analysis and quantitative PCR of cell cycle regulator genes revealed that both unstimulated and BαP-stimulated AHR reduced E2F-driven transcription and altered expression of cell cycle regulator genes, thus increasing the percentage of cells in G_0_/G_1_ phase and slowing the proliferation rate of GH3 cells. Co-immunoprecipitation confirmed the interaction between AHR and retinoblastoma (Rb1) protein supporting this as a functional mechanism for the observed reduction. Endogenous Ahr reduction using silencing RNA confirmed the tumour suppressive function of the Ahr. These data support a mechanistic pathway for the putative tumour suppressive role of AHR specifically in PA, possibly through its role as a cell cycle co-regulator, even in the absence of exogenous ligands.

## Introduction

Pituitary adenomas (PA) represent the commonest cranial neoplasms and vary in size, type and aggressiveness. Clinically relevant PAs result in symptoms due to hormonal hypersecretion, intracranial mass effects or secondary hypopituitarism and locally occur with an average prevalence of approximately 76/100,000 (Gruppetta *et al.* 2013). The vast majority of PAs are sporadic in origin with only a small number of significant genetic mutations identified in relatively rare familial cases and endocrine syndromes. The heterogenous nature of PAs poses challenges in the elucidation of the molecular mechanisms driving their formation and progression (Asa *et al*. 2017). This contrasts significantly with current knowledge in relation to malignant tumours.

Certain key pathways have already garnered a great deal of attention, including the cAMP secondary messenger signalling pathway, the Wnt signalling pathway and the pI3K/Akt signalling pathways (Chambers *et al.* 2013, Formosa & Vassallo 2014, Monsalves *et al.* 2014, Peverelli *et al*. 2014). However, even collectively, these molecular pathways do not cater for the entirety of the mechanisms that are responsible for the development and/or progression of PAs, making our understanding of the disease as yet limited. Several studies using high-throughput techniques including microarray studies and large-scale sequencing have identified a number of interesting pathways (Moreno *et al.* 2005, Morris *et al.* 2005, Evans *et al.* 2008, Jiang *et al.* 2010, Newey *et al.* 2013, Valimaki *et al.* 2015). In an attempt to uncover more molecular mechanisms driving Pas, we screened RNA profiles expressions of local tumours to uncover common altered pathways among a heterogeneous set of PAs. A consistently altered pathway observed implicated xenobiotic signalling.

The AHR is a ligand-activated transcription factor containing the basic helix-loop-helix (bHLH)/PAS domain mediating the response to a variety of environmental toxins (Burbach *et al.* 1992). Unliganded AHR is maintained in the cytoplasm bound to a chaperone complex together with HSP90, p23 protein and the aryl hydrocarbon receptor-interacting protein (AIP). Upon ligand binding, the AHR disengages and translocates to the nucleus where it binds to the aryl hydrocarbon nuclear translocator (ARNT). Together with a number of co-activators and co-repressors, AHR forms a transcription complex on consensus DNA sequences called xenobiotic response elements (XRE) located upstream of the promoters of AHR target genes such as the cytochrome P450 genes (CYP1A1 and CYP1B1) (Davarinos & Pollenz 1999, Whitlock 1999). AHR signalling has been shown to be functionally active in pituitary cells, which respond rapidly to exogenous ligands (Huang *et al.* 2002).

The AHR is constitutively active and plays a significant role in cell homeostasis in a number of organs in the absence of any exogenous ligand (Singh *et al.* 1996, Crawford *et al.* 1997, Chang & Puga 1998, Murray *et al.* 2005). AHR knockout mice have decreased liver size, decreased body weight and reduced reproductive potential. AHR also directly effects cell cycle progression and differentiation in the absence of any exogenous ligand in several cell lineages (Bauman *et al.* 1995, Ma & Whitlock 1996, Levine-Fridman *et al.* 2004, Marlowe *et al.* 2004).

There are conflicting reports postulating both tumour suppressive and oncogenic roles for AHR depending on cell and context specificity. Most data so far regarding the involvement of AHR in cancer have been obtained from malignant forms of the disease indicating both a cancer promoting role for AHR (Andersson *et al.* 2002, Moennikes *et al.* 2004, Feng *et al.* 2013) or a tumour suppressive role in cancer (Schmidt *et al.* 1996, Gonzales *et al.* 1998, Ito *et al.* 2004, Mulero-Navarro *et al.* 2006, Fritz *et al.* 2009, Fan *et al.* 2010, Spink *et al.* 2013). However, no direct evidence has been found as yet linking the functional role of AHR in pituitary tumour formation or behaviour. Investigation in PAs has focused mainly on the role of AHR in relation to the AIP. Germline mutations of AIP have been found to increase susceptibility to familial cases of PAs (Vierimaa *et al.* 2000). Lecoq *et al.* (2016) showed that AIP mutations do not alter AHR expression but can downregulate the expression of AHR target genes, thereby effecting AHR transcriptional activity. Reports documenting detailed analysis of this and possibly other mechanistic pathways involved in pituitary tumourigenesis are to date lacking.

The aim of our study was therefore to identify novel pathways involved in pituitary tumour formation by microarray analysis of PAs from Maltese patients compared to a pooled control sample and subsequently to functionally analyse such pathways using *in vitro* models.

## Materials and methods

### Tumour collection and characterization

Tumours were collected from 31 patients undergoing trans-sphenoidal surgery at Mater Dei Hospital, Malta (19 non-functioning PA, 8 GH-secreting PA, 2 PRL-secreting PA, 2 ACTH-secreting PA). Informed consent was obtained from all patients. Samples were collected in RNAlater (Life Technologies) and stored at −20°C until processing. Data on the clinical and biochemical hormone profiles and as well as standard immunohistochemistry for anterior pituitary hormones were used for characterization of the adenomas. Patients’ details are summarized in Table 1. Control human pituitary mRNA was obtained from Clontech and contained pooled RNA from 39 male/female Caucasians aged 20–60 years.

**Table 1:** Patient characteristics of tumour RNA used for microarray analysis.

**Patient**	**Sex**	**Age at diagnosis**	**Tumour type**	**Tumour diameter** (mm)	**Treatment**	**Invasive**
1	F	62	TSH/prolactin secreting	24	Octreotide	No
2	F	40	Acromegaly	20		Yes
3	M	52	Acromegaly	10		No
4	M	60	NFPA	20		No
5	F	79	NFPA	NA		Yes
6	M	47	NFPA	25	Bromocriptine	Yes
7	M	48	NFPA	36		Yes
8	F	54	NFPA	13		Yes

### RNA extraction and quantitative PCR (qPCR)

Pituitary samples were removed from RNAlater and placed at −80°C overnight. Cells were disrupted using a mortar and pestle and homogenized using Qiashredder (Qiagen). RNA was extracted using a standard protocol from RNeasy Mini extraction kit (Qiagen). RNA extraction from GH3 cells was carried out using the RNeasy Mini kit 48 h after transfection with expression vectors and 24 h after treatment with activating ligand in 6-well plates.

cDNA synthesis for qPCR was carried out with 1 μg of RNA and random hexamers using the GoScript Reverse transcription system (Promega). Details of qPCR conditions and list of primers used can be found in the Supplementary section (see section on supplementary data given at the end of this article). qPCR on RNA from 31 tumours samples collected locally was used to verify results obtained by microarray. Subsequently, qPCR on GH3 cells was carried out to study the effects of AHR over-expression and knockdown on target gene transcription.

### Microarray and data analysis

mRNA profiling of the eight tumour samples and control pituitary RNA was carried out using the HuGene 1.0 ST Array (Affymetrix) at Erasmus Medical Centre, Erasmus University, Rotterdam, the Netherlands. Data analysis was carried out using the GeneSpring 11.0 software (Agilent Technologies, with comparison between all tumours vs controls, non-functional tumours vs controls, functional tumours vs controls and functional vs non-functional tumours). A two-fold difference was used as a cut-off for significant results.

Data were analysed through the use of QIAGEN’s ingenuity pathway analysis (IPA, QIAGEN Redwood City, www.qiagen.com/ingenuity). The most significant pathways were identified based on right-tailed Fisher’s exact test.

### Cell culture and in vitro analysis

*In vitro* analysis was carried out to analyse the effect of AHR signalling on GH3 rat sommatolactotroph pituitary cells. GH3 cells were cultured in DMEM with 10% FBS and 1% antibiotics (Sigma). To study the influence of AHR signalling, GH3 cells were transfected with AHR expression vector in pcDNA3, generously donated by Prof. Anette Duensing, University of Pittsburgh Cancer Institute, Pittsburgh, USA. Activation of AHR was achieved using benzo (alpha) pyrene (BαP) (Sigma) at concentrations varying from 0.1 µM to 10 µM dissolved in DMSO and diluted in water. Knockdown of endogenous Ahr was achieved using commercial siRNAs (Dharmacon) and optimized with Lipofectamine RNAiMAX transfection agent.

### Proliferation assays

GH3 cells were seeded in 96-well plates at 10,000 cells per well. After 24 h, cells were transfected with varying quantities of AHR expression vector using Fugene HD (Promega) transfection reagent at a 3:1 (Fugene:DNA) ratio according to manufacturer’s instructions. For knockdown experiments, GH3 cells were transfected with 0.5, 1 or 2 pmol of rat Ahr siRNA or control (negative) siRNA (Dharmacon, USA) using Lipofectamine RNAiMAX (Invitrogen) using the manufacturer’s instructions. Cells were then left in the incubator for 24–72 h post transfection. MTT readings were taken at 24-h time intervals up to 96 h post transfection using the Mithras LB 940 (Berthold Technologies, Bad Wildbad, Germany). Five repeats per treatment were used and experiments were carried out in triplicate.

### Luciferase reporter gene activity

Dual luciferase assays reporter assays were used to assess the activity of AHR in GH3 cells. The transcriptional activity on the xenobiotic response element (XRE) was assessed using an XRE-driven reporter expression vector containing 3 copies of the Cyp1a1 XRE kindly donated by Prof. Albert Braeuning (University of Tuebingen, Germany). The effect of AHR on E2F transcriptional activity was analysed using an E2F-driven reporter plasmid containing three E2F-binding sites kindly donated by Prof. Alvaro Puga (University of Cincinnati, OH, USA).

For reporter gene activity, GH3 cells were seeded in 2F4-well plates at 50,000 cells per well. After 24 h, transfections with expression vectors and/or silencing RNA were carried out in accordance with the manufacturer’s instructions. Transfection mixtures contained 0.4 μg AHR vector or empty vector (pcDNA3 only), 0.4 μg reporter plasmid (XRE or E2F reporter gene) and 0.01 µg pRL-TK (renilla expression vector) per well. For knockdown experiments, 5 pmol of Ahr siRNA or control siRNA replaced the AHR/EV expression vectors in the transfection mixtures and RNAiMAX reagent was used instead of FuGENE. 24 h after transfection, BαP was also added at the required concentration. After another 24 h, cells were lysed with 100 µL passive lysis buffer and dual luciferase activity was read using the Dual Luciferase Reporter Assay (Promega) in the TD-20/20 Luminometer (Turner Designs, San Jose, CA, USA). Each treatment was carried out in triplicate, and experiments were replicated 3 times.

### Co-immunoprecipitation and Western blot

Co-immunoprecipitation (co-IP) was carried out on GH3 cells treated with either vehicle (DMSO) or 1 μM BαP for 1 h prior to lysis in whole cell lysis buffer. Co-IP was also carried out on GH3 cells transfected with AHR expression vector or Ahr siRNA and lysates where taken 48 h after transfection. Co-IP was carried out using the Universal Magnetic Co-IP kit (Active Motif, Carlsbad, CA, USA) using the manufacturer’s instructions. 500 µg of whole cell lysate was incubated with 2 µg of Ahr antibody (RPT1, Novus Biologicals, Littleton, CO, USA) for 4 h on ice prior to precipitation with magnetic beads. 20 µg of eluted protein was then analysed by standard Western blot for the presence of the precipitated endogenous Ahr (RPT1 antibody) and the co-immunoprecipitated Rb protein using Rb1 antibody (1F8, Novus Biologicals) for detection. Detection was carried out using anti-mouse secondary antibody (IRDye 800CW, Li-Cor Biotechnology, Lincoln, NE, USA) and read on the Odyssey Imaging System (Li-Cor Biotechnology). β-Actin antibody (AC-15, Novus Biologicals) was used to verify the success and specificity of co-immunoprecipitation. Similar protocol was used to verify the success of transfection with AHR vector and knockdown of endogenous Ahr with siRNA.

### Cell cycle analysis

Cell cycle analysis was carried out on GH3 cells in 24-well plates under the same experimental conditions used for the luciferase reporter assays except that only one vector or siRNA was transfected at any experimental condition. Prior to addition of BαP, the medium was replaced with fresh DMEM with no serum or antibiotics, and after 6 h, serum was added to synchronize the cells. After 24 h, cells were fixed in cold 70% ethanol overnight, washed with PBS and re-suspended in 500 μL of RNase solution (Sigma) and left for 30 min at 37°C. The cells were stained with 200 μL 50 μg/mL propidium iodide (Sigma) prior to flow cytometry, which was carried out using FACSCalibur (BD Biosciences, USA). Data were analysed using CellQuest Pro (BD Biosciences). Experiments were carried out in triplicate and replicated three times.

### Statistics

All data were analysed using SPSS Statistics, version 17 (IBM). Kolmogorov–Smirnov test was carried out on all the raw data to determine the normal distribution of the data. For the proliferation assays, ANOVA analysis was used, while for the luciferase assays, real-time PCR and cell cycle analysis, pairwise *t*-tests (for parametric data) or Wilcoxon *t*-test (for non-parametric data) were used. Statistical significance was set at the 5% confidence level.

## Results

### Microarray analysis and confirmation by quantitative PCR

Microarray analysis carried out on 8 tumours and a pooled control RNA revealed significant differences. Comparison by gender and tumour invasiveness revealed little or no difference in mRNA expression profiles of tumours. Comparison of controls vs all tumours revealed a total of 1409 differently expressed genes, of which 842 genes were downregulated and 567 were upregulated. Expression profiles of the different tumour types were also compared to each other and to controls separately. Additionally, one tumour (patient 8) characterized biochemically and immunohistochemically as a non-functional adenoma had a RNA expression profile resembling a functional tumour when unsupervised clustering was carried out as shown below. Upon further patient follow-up and biochemical investigation, the patient was indeed found to have a functional adenoma. In subsequent analyses, this tumour was therefore grouped with the functional adenomas.

**Figure 1:**
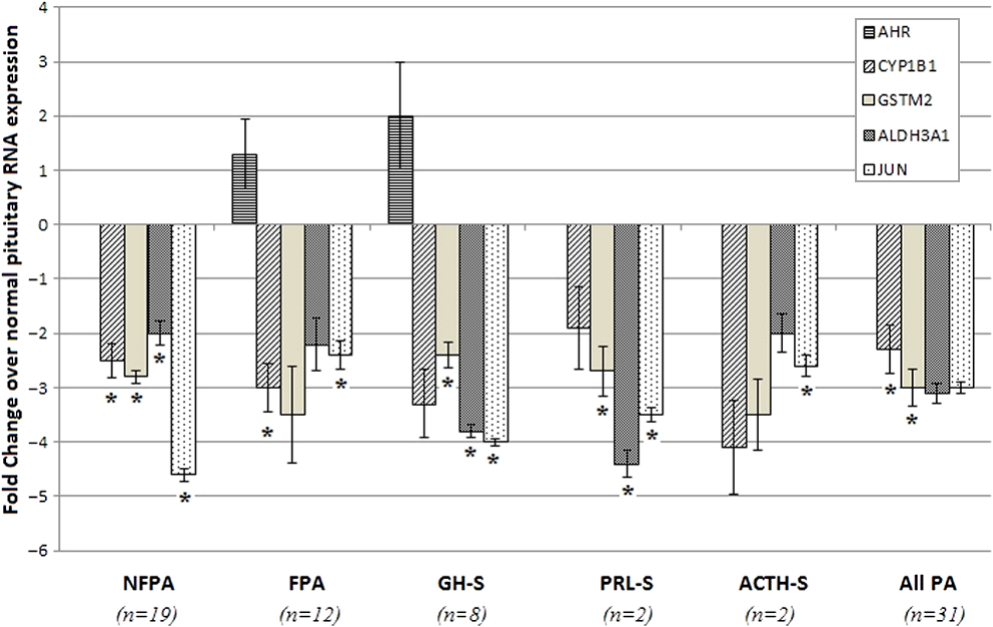
qPCR of target genes to confirm data obtained by microarray analysis. Genes with de-regulated expression as compared to normal pituitary were analyzed using GAPDH and EMC7 as housekeeping genes. (CYP1B1, cytochrome P450 1B1, GST; gluthionine S-transferase, ALDH3A1; aldehyde dehydrogenase family 3, A1, JUN; Jun oncogene, NFPA; non-functioning PA, FPA; functional PA, GH-S; growth hormone secreting PA, PRL-S; prolactin-secreting PA, ACTH-S, adrenocorticotropic hormone-secreting PA). qPCR experiments were done in triplicate and error bars indicate standard error. (*P<0.05).

qPCR was subsequently carried out on 31 samples including the 8 patients used for microarray analysis. A number of genes found to be deregulated by microarray analysis and related to the AHR signalling pathway were selected. qPCR confirmed the findings observed by microarray analysis (Fig. 1). AHR expression levels *per se* were not significantly altered, while xenobiotic target genes identified by microarray, were found to be significantly reduced in the 31 tumours analysed, thereby confirming the findings of the microarray and pathway analyses regarding the downregulation of AHR signalling.

### Ingenuity pathway analysis (IPA)

The IPA software was used to analyse the list of de-regulated gene expressions and to identify pathways and cellular mechanisms that are altered in PAs as reflected by their expression profiles. IPA uses a vast database of gene functions and interactions based on data from human, rat and mouse studies.

Among the many pathways identified by the software, one of the most prominent and recurring pathways identified during each analysis was the aryl hydrocarbon receptor (AHR) signalling pathway. This pathway was significantly altered in all comparisons, whether comparing all tumours to control or specific tumour types alone to controls, with a *P* value of less than 0.001 in all analyses. Supplementary Figure 1 indicates part of the AHR signalling pathway, which was found to be de-regulated in all PA vs controls. Although the AHR itself was not found to be significantly differentially expressed, the cut-off level of a two-fold difference implies that small changes might be missed. However, several downstream AHR transcriptional targets, such as *CYP1B1*, *ALDH* and *GST* genes were found to be significantly reduced with more than two-fold differences observed between tumours and controls.

### In vitro analysis of AHR activity in GH3 cells

**Figure 2:**
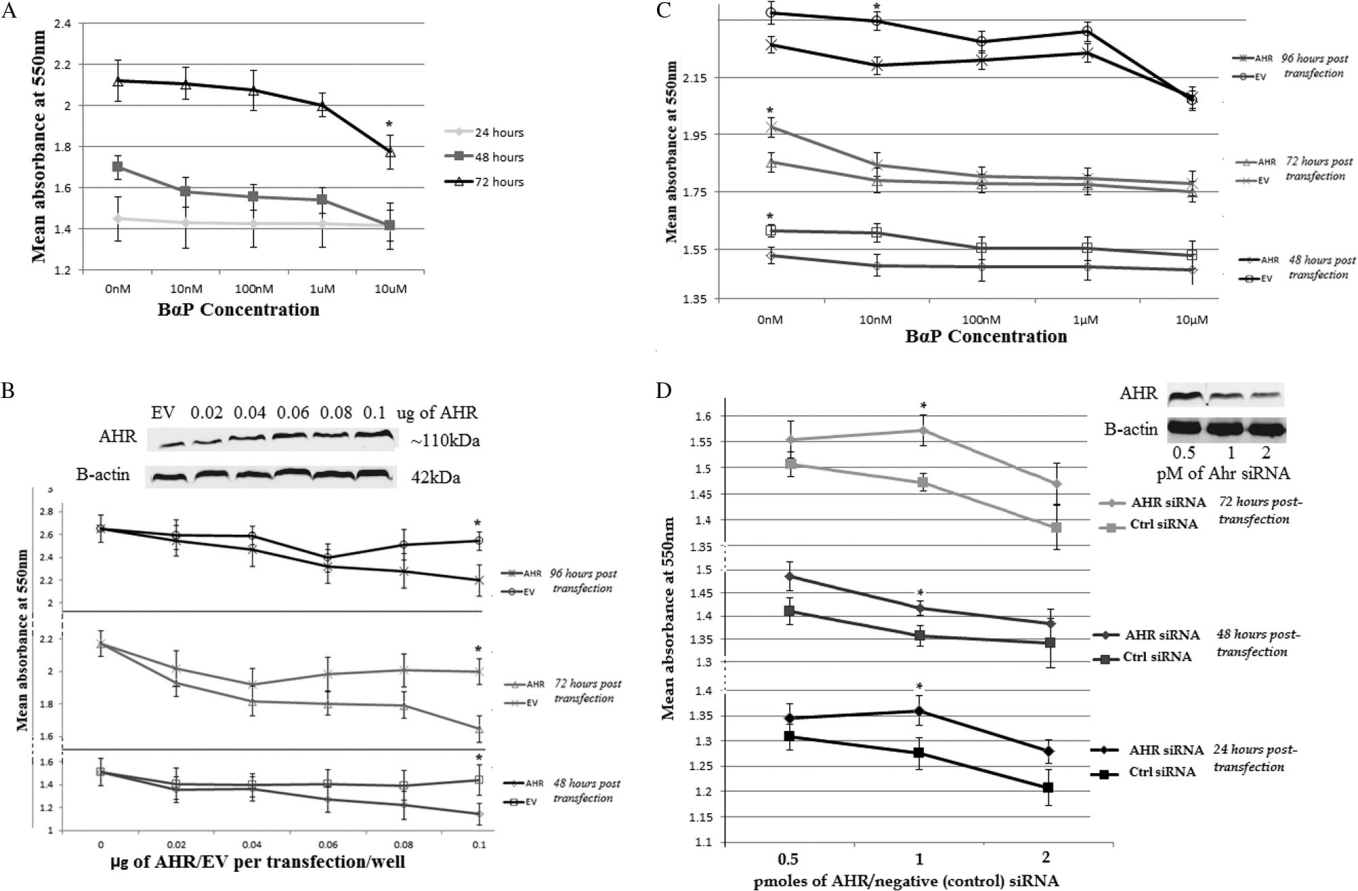
Proliferation (MTT) assays were carried out to study the role of AHR signaling on GH3 cell proliferation at different time points. (A) GH3 cells treated with BαP at different concentrations over three days. (B) GH3 cells transfected with increasing amounts of AHR/EV (pcDNA3) expression vectors and analyzed at three different time points from transfection. Insert shows increase in expression of transfected AHR with western blot using B-actin as loading control (C) GH3 cells transfected with AHR or EV plasmid and treated with BαP at different concentrations 24 hours after transfection and assayed at different time points post-transfection. (D) GH3 cells were transfected with Ahr or Control (Ctrl) siRNA at different concentrations and proliferation measurements taken at 3 time points. Insert shows a western blot reflecting the efficiency of Ahr silencing using siRNA. Experiments were done in repeats of 5 per treatment and each experiment was repeated at least three times. (*P< 0.05, error bars represent standard error).

Given the downregulation of AHR signalling demonstrated by microarray and IPA, *in vitro* analyses using the established GH3 cell line were performed to investigate the functional role of the AHR in PA. Proliferation analysis was carried out using MTT assays with BαP, a potent ligand of the AHR, as an activator. Endogenous Ahr activation by BαP alone did not alter cell proliferation significantly except at a concentration of 10 μM after 72 h of treatment, an effect likely to be due to toxicity of the agent as observed microscopically (Fig. 2A).

In order to further investigate the role of AHR in mediating cell proliferation and function, it was decided to analyse the effect of over-expressing AHR in GH3 cells. As observed in Fig. 2B, increasing AHR resulted in a dose-dependent decrease in cell proliferation with a significant decrease observed at 0.1 μg of transfected AHR vector when compared to the same dose of empty vector at each time point and in the absence of exogenous ligand. The combination of both AHR overexpression and BαP ligand was also tested (Fig. 2C). At all time points, GH3 cells overexpressing AHR without BαP treatment all had significantly lower proliferation when compared to cells treated with empty vector alone. Addition of BαP only resulted in a significant reduction in proliferation when compared to similarly treated cells with empty vector, at very low concentrations of 10 nM and only at two time points. Further increase in BαP concentration did not result in any significant difference in proliferation between cells overexpressing AHR or empty vector.

In order to confirm our findings, knockdown of endogenous Ahr using silencing RNA was also carried out using three concentrations of the siRNAs and at three different time points. As observed in Fig. 2D, GH3 cells with reduced endogenous Ahr expression had higher proliferative rates than their control counterparts at all time points and concentrations, with significant differences observed at 1 pmol of siRNA transfected per well. Higher concentration of siRNA resulted in reduced proliferation probably due to the toxic effect of higher doses of the transfection reagent.

### AHR signalling in GH3 cells

Since BαP treatment on GH3 cells and GH3 cell overexpressing human AHR had little effect on cell proliferation, we wanted to verify whether xenobiotic AHR signalling was indeed occurring within the GH3 cells. Luciferase reporter assay using an XRE-driven luciferase gene showed a significant increase in luciferase activity only in the presence of BαP activating ligand and in a dose-dependent manner (Supplementary Fig. 2A). No activation was observed at 10 nM since this concentration is too low to achieve significant activation (De Waard *et al.* 2008) while 10 μM BαP proved too toxic to the cells. Knockdown of endogenous Ahr attenuated the XRE-driven luciferase activity, thereby showing the direct functional impact of Ahr and its activating ligand on XRE promoter activity.

Secondly, quantitative PCR was used to measure the expression levels of Ahr target genes in the GH3 cells to ascertain that the transfected AHR could functionally activate transcription in these cells. Real-time PCR of three Ahr target genes, *Cyp1a1*, *Cyp1b1* and *Aldh3a1*, was carried out (Supplementary Fig. 2B). Treatment with BαP alone was able to significantly stimulate *Aldh3a1* gene expression only while transfection with AHR expression vector and treatment with exogenous activator was able to significantly induce expression of all three target genes showing the functional ability of AHR to activate the transcription of target genes even in rat GH3 cells. Similarly, knockdown of endogenous Ahr once again attenuated this effect and rescue with human AHR by transfection was able to restore downstream Ahr target expression in response to ligand (Supplementary Fig. 2B).

### AHR-Rb co-immunoprecipitation and effect on cell cycle regulators

**Figure 3:**
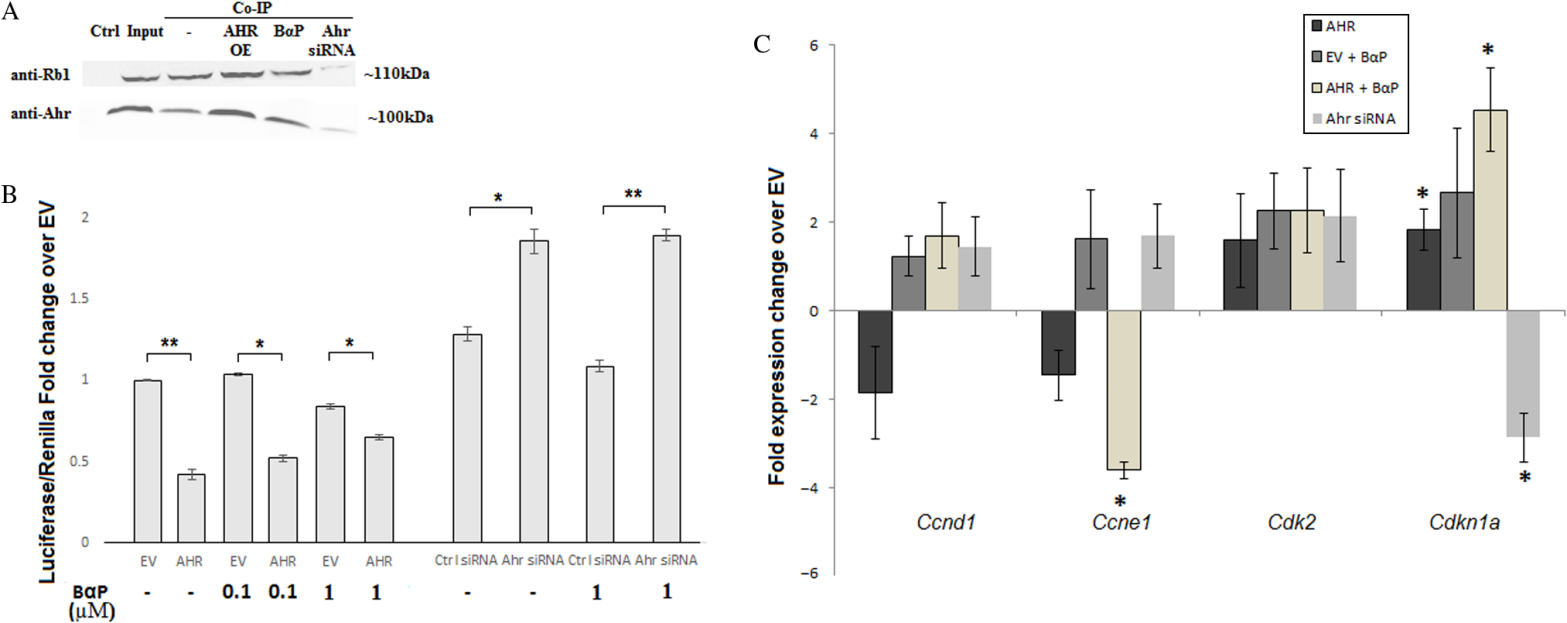
Co-immunoprecipitation of Ahr and Rb1 in Gh3 cells and the effect of AHR over-expression and exogenous stimulation on cell cycle regulators in GH3 cells. (A) Co-IP was carried out in GH3 cells and western blot with 20μg of cell lysate or precipitated proteins was run using anti-Rb1 and ant-Ahr antibodies on a negative control with lysate run through Ig beads with no antibody (Ctrl), the whole cell lysate prior to precipitation (input) and Co-IP using anti-AHR for pull down. Lane 1 of the co-IP represents untreated cells, lane 2 contains the precipitate from GH3 cells over-expression AHR, lane 3 from GH3 cells treated with 1µM BαP prior to lysis and lane 4 with lysate from GH3 cells with knocked down Ahr protein. (B) GH3 cells were co-transfected with AHR or EV together with an E2F-driven luciferase reporter plasmid and treated with no BαP (-), 100nM or 1µM BαP for 24 hours prior to lysis and luciferase readings. For knock down experiments, GH3 cells were transfected with reporter plasmid and Ahr or control (Ctrl) siRNA) for 24 hours prior to luciferase reading. (C) GH3 cells were transfected with either AHR/EV or Ahr siRNA and treated with 1μM BαP for 24 hours prior to lysis and RNA extraction. Experiments were repeated three times and luciferase studies were done in triplicate while qPCR readings were done in duplicate and repeated three times. (*P<0.05, **P< 0.01, error bars indicate standard error).

Given the reported direct and indirect interaction of AHR with a number of cell cycle regulators such as Rb and CDNKs (Kolluri *et al.* 1999, Puga *et al.* 2000), we wished to investigate whether AHR overexpression and activation would have the same effects in pituitary cells as those reported in other tissues. The interaction between AHR and Rb proteins has been documented in a number of cell types resulting in prolonged activity of Rb and reduction in E2F transcriptional activity normally required for cell cycle progression (Feng *et al.* 2013). In order to verify whether this interaction was present in our cell line, co-immunoprecipitation of endogenous Ahr was carried out. As observed in Fig. 3A, isolation of Ahr from the cell lysate of GH3 cells also precipitated Rb protein with the addition of BαP ligand having little effect on the amount of Rb protein co-precipitated. Overexpression of AHR resulted in slightly increased Rb precipitation while silencing of endogenous Ahr reduced the amount of precipitated Rb considerably.

We therefore proceeded to analyse the effect of AHR on E2F-driven transcription using an E2F-luciferase reporter expression vector. Overexpression of AHR showed a marked decrease in E2F-driven luciferase expression in cells while Ahr silencing resulted in an increase in E2F-driven luciferase activity (Fig. 3B). Treatment with BαP did not have any significant effect on E2F-driven transcription at any concentration, thus supporting the hypothesis that the reduction in E2F-driven luciferase was achieved through Ahr protein expression alterations without the need for an exogenous activating ligand in GH3 cells.

The expression of particular cell cycle regulators that are known to be affected by AHR was also analysed. Cdkns, cyclins and cdks were reported to be differentially expressed *in vitro* as a result of AHR signalling activity (Levine-Fridman *et al.* 2004, Marlowe *et al.* 2006, Pang *et al*. 2008, Tomblin & Salisbury 2014). Real-time PCR for the specific genes is shown Fig. 3C below. Cyclin D1 (*Ccnd1*) expression was not affected while Cyclin E1 (*Ccne1*) expression was significantly reduced by overexpressed AHR treated with ligand. *Cdkn1a* (p21) gene expression was significantly overexpressed in GH3 cells overexpressing AHR only, and this effect was further amplified by addition of BαP activating ligand. Knockdown of Ahr by mRNA silencing only significantly reduced *Cdkn1a* expression, a reversal of the effect of AHR overexpression. In conclusion therefore, AHR activity in GH3 cells significantly altered the expression of at least two important inhibitors of cell cycle progression.

### Cell cycle genes expression in PA

**Figure 4:**
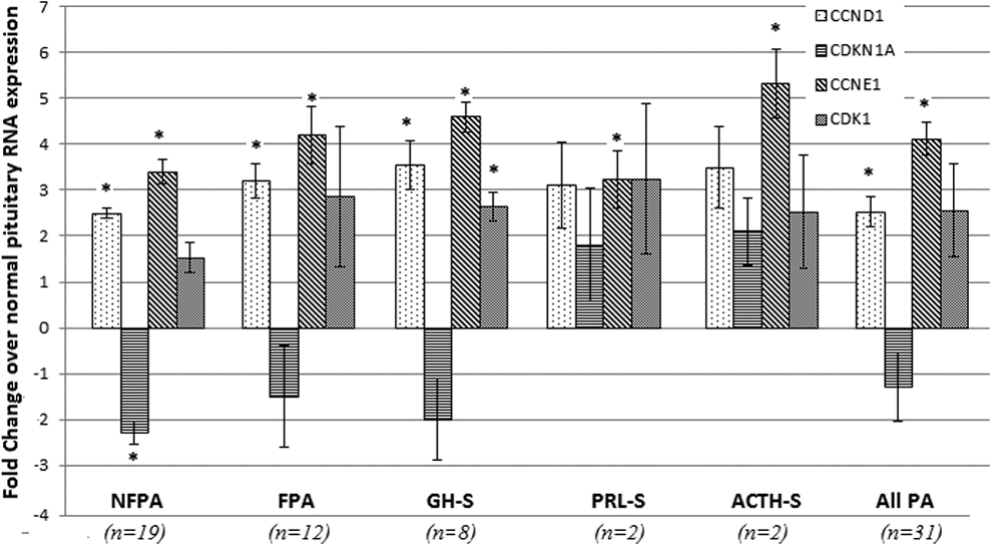
Gene expression analysis of specific cell cycle regulator genes in 31 pituitary adenomas, divided by tumour type and shown as fold changes over control pituitary RNA. qPCR readings were done in duplicate and repeated three times. (*P<0.05, error bars indicate standard error).

Expression in CDKs and CDKNs was also altered in the pituitary tumour RNA from the 31 patients. Microarray analysis revealed the *CDKN2A* and *CDKN1A* genes to be significantly repressed in all tumour types vs controls while the *CDK2* gene was upregulated in the all tumours vs controls and *CDK6* was also upregulated in GH-secreting tumours vs controls (data not shown). These results were tested by qPCR (Fig. 4) and gene expression of several other cell cycle regulators was also analysed. No significant differences in *CDKN1B* and *CDK2*, *6* gene expression (data not shown) was observed in the tumours vs control RNA. Cyclin E1 (*CCNE1*) expression was significantly higher in all tumour types vs controls while Cyclin D1 (*CCND1*) gene expression was higher in all tumour types except prolactinomas and ACTH-secreting tumours when compared to control RNA. *CDKN1A* (p21) gene expression was significantly lower in non-functioning tumours vs controls while *CDK1* expression was high in all tumour types but only reached statistical significance in GH-secreting tumours. Lack of statistical significance for the prolactinomas and ACTH-secreting tumours is likely due to the small number of samples available.

### AHR effects cell cycle progression

**Figure 5:**
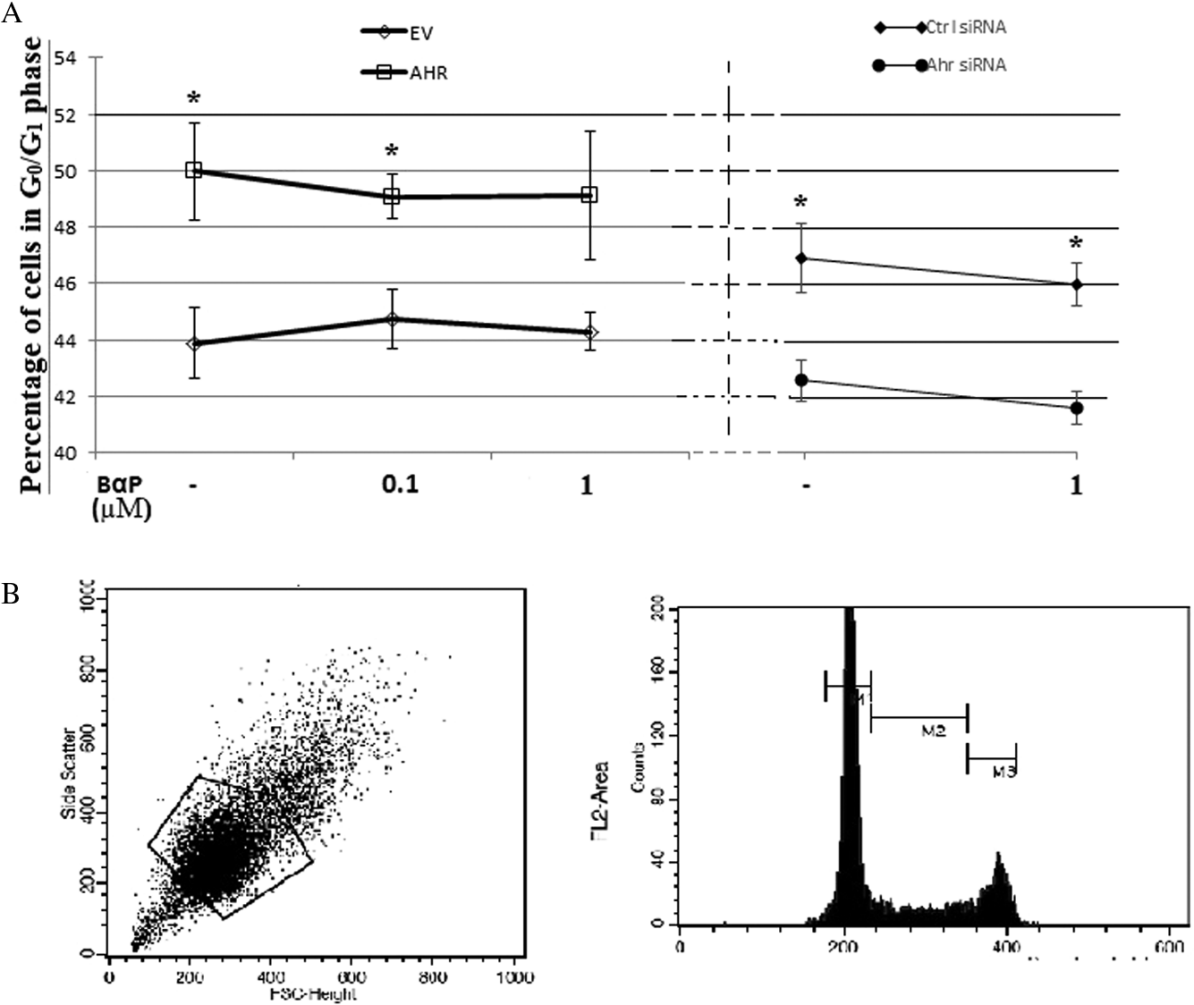
Cell cycle analysis was carried out on GH3 cells transfected with AHR/EV or Ahr/Ctrl siRNA and treated with vehicle or BαP for 24 hours prior to fixation and flow cytometry. (A) Percentage of GH3 cells in G0/G1 phase in differently treated cells. Cell cycle analysis was done in triplicate and experiment was repeated three times using synchronized cells. (B) Cell Quest Pro software was used to gate the cell population and only gated cells were used for the final cell cycle analysis. Areas designated as belonging to G0/G1 (M1), S phase (M2) and G2 phase were assigned as shown. (*P<0.05, error bars represent standard error).

AHR activity has been shown to affect the cell cycle both in the absence and presence of activating ligands in cells lines of mostly epithelial, hepatic or breast origin. Data from GH3 cells overexpressing AHR demonstrate that both stimulated and unstimulated AHR can reduce E2F transcriptional activity and expression of cell cycle regulators in GH3 cells. Therefore, the direct effect on cell cycle progression was further analysed using propidium iodide and flow cytometry. As observed in Fig. 5 below, GH3 cells overexpressing AHR had a significantly higher percentage of cells in G_0_/G_1_ phase when compared to cells transfected with an empty vector. Treatment with 100 nM and 1 μM exogenous BαP did not alter the percentage of cells in G_0_/G_1_ significantly. Conversely, GH3 cells with reduced expression of endogenous Ahr had lower percentage of cells in G_0_/G_1_ phase.

## Discussion

The role of the AHR in carcinogenesis has been the subject of debate since it appears to be able to act both as an oncogene and a tumour suppressor in different cell types and contexts (Marlowe & Puga 2005). However, most cases of oncogenic AHR activity involve malignant forms of cancer, which differ significantly from pituitary tumour behaviour, which is benign in nature. Very few studies have looked at the impact of AHR signalling on PA. AHR expression was found to be altered in one study (Jaffrain-Rae *et al.* 2009) with reduced immunohistochemical expression of AHR in pituitary tumours as compared to normal controls. AHR was also found to be under-expressed in invasive adenomas and those tumours with demonstrated AIP mutations. Another study by the same group also revealed a significant negative correlation between AHR expression and pituitary tumour size and suprasellar extension indicating again that tumours with high AHR exhibited less aggressive behaviour, thereby indicating a possible growth advantage obtained by reducing AHR expression in tumours (Jaffrain-Rae *et al.* 2013). Similarly, protein data mining by Zhan and Desiderio (2010) using IPA on several proteomic expression data identified the AHR signalling pathway as a significantly altered pathway in almost all of their analyses, with this pathway featuring repeatedly in the top 10 significantly altered pathways although no indication was given whether the pathway was activated or repressed in PAs. Additionally, one microarray study carried out on 5 prolactinoma samples identified members of the xenobiotic signalling pathway to be significantly downregulated but failed to elaborate further on these findings (Jiang *et al.* 2010). Hence, evidence for a tumour-suppressive role in PA already existed, based on previous studies. This paper is the first to map out a mechanistic pathway for AHR in pituitary tumourigenesis using both *in vivo* material from human pituitary tumours and *in vitro* analysis.

In contrast to other studies involving AHR in cancer biology, this study was able to prove that AHR may act on the cell cycle even in the absence of an exogenous activating ligand, thereby suggesting a mechanism of action independent of its role as a xenobiotic receptor. In this study, BαP was used as an exogenous AHR agonist in preference to several other possible ligands such as TCDD. BαP is a polycyclic aromatic hydrocarbon and considered a potent activator of AHR (Matsunawa *et al.* 2009), which is what was required by this study and achieved as indicated by the successful activation of DRE-driven reporter activity and AHR target gene expression. Conversely, a more common agonist, TCDD, is incompletely metabolized by xenobiotic enzymes, which could lead to more off-target effects (Bohonowych & Denison 2007). Owing to the complex biology of AHR, different agonists may result in different AHR-driven activation pathways (Hockley *et al.* 2007, Harper *et al.* 2013, Lowe *et al.* 2014) and therefore the use of any agonist has to be taken with adequate consideration regarding this fact.

*In vitro* studies using GH3 cells were used to study the functional role of the AHR in pituitary tumours. GH3 cells are of rat origin and are currently the most commonly used *in vitro* model of functional secreting tumours since they secrete both growth hormone and prolactin. GH3 cells possess functional AHR, although at low expression levels and do respond to both classical and novel agonists through increased expression of xenobiotic metabolic enzymes (Moran *et al.* 2012, Long *et al.* 2013, Lecoq *et al.* 2016). Proliferation analysis revealed the tumour suppressive ability of the AHR with increasing AHR expression resulting in a relative reduction in GH3 cell proliferation. Silencing of endogenous Ahr similarly resulted in an increase in GH3 proliferation while the addition of exogenous ligand did not influence either scenario significantly indicating distinct roles of AHR as a xenobiotic receptor and as a regulator of proliferation.

Review of the literature supported a possible role of AHR on the cell cycle independent of its function as a xenobiotic receptor. Studies regarding the mechanism(s) by which AHR regulates cell proliferation demonstrated direct interaction with the retinoblastoma (Rb) protein in 5L hepatoma and MCF-7 breast carcinoma cells (Ge & Elferink 1998, Puga *et al.* 2000). The Rb protein in its hypophosphorylated state inhibits the G1/S transition by binding to and repressing E2F transcription factors, thus preventing the transition to the S phase (Dyson 1998). AHR binds to Rb and maintains it in its active hypophosphorylated state (Puga *et al.* 2000). AHR has also been reported to activate transcription of the cyclin-dependent kinase inhibitor *CDKN1B* (p27) gene, a tumour suppressor that inhibits cyclin–cyclin-dependent kinase (CDK) interactions and maintains Rb in its hypophosphorylated state (Kolluri *et al.* 1999).

Our results demonstrate that AHR overexpression reduces cell proliferation and inhibits cell cycle progression in GH3 cells. Using co-immunoprecipitation, the physical interaction between Ahr and Rb in GH3 cells was confirmed in accordance with other studies carried out in different cell contexts (Ge & Elferink 1998, Marlowe *et al.* 2004). Additionally, our results also indicate that AHR overexpression is able to increase expression of CDKIs, particularly p21, and reduce expression of cyclin E1, two important mediators of the cell cycle. Jackson and coworkers (Jackson *et al*. 2014) postulate that AHR can directly transactivate expression of the p21 gene through a non-consensus XRE. Therefore, we postulate that AHR, acting through its both or either of these two mechanisms, that is, the interaction with Rb and the ability to increase expression of CDKIs, is able to reduce E2F-driven cell cycle progression, an effect that is not altered by the presence of an exogenous agonist. In fact, addition of ligand appears to hinder AHR-mediated cell cycle inhibition, allowing the hypothesis that an activating ligand might sequester AHR towards its xenobiotic function and inhibit its ability to repress cell cycle progression. These results are in agreement with those reported by Moran and coworkers (Moran *et al*. 2012) in which GH3 cells treated with AHR ligand β-naphthoflavone failed to show any significant difference in proliferation or cell cycle progression, indicating that activation of xenobiotic AHR signalling alone does not alter pituitary cell proliferation. Notwithstanding, one can never exclude completely the presence of endogenous AHR ligands which, although are generally of low affinity, may still activate the AHR sufficiently (Opitz *et al.* 2011).

**Figure 6:**
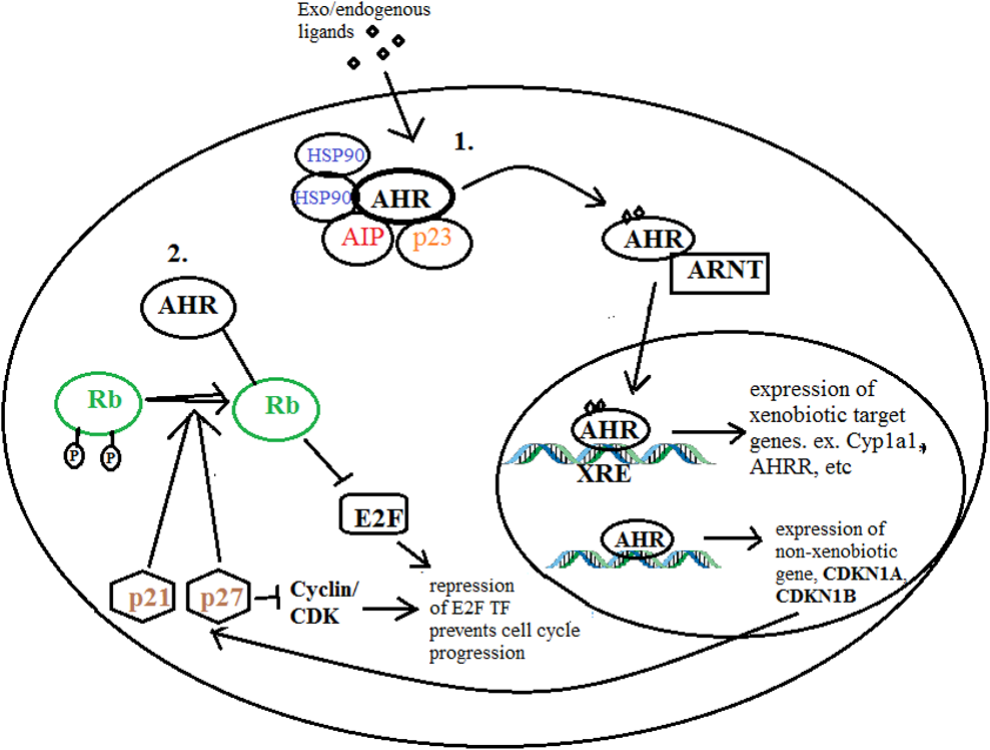
Illustration of AHR activity in pituitary cells. The AHR may act through two pathways in the pituitary cells which can either be distinct in activity or influence each other by sequestering AHR protein. Pathway 1 illustrates the standard canonical xenobiotic signaling by AHR which is activated by exogenous or endogenous ligands to translocate to the nucleus together with ARNT and transcribe target genes such as CYP1A1, AHR among others. The second pathway involves the influence of AHR on cell cycle regulators. Through its interaction with Rb protein, AHR is able to maintain it in its hypo-phosphorylated state where it inhibits E2F activity and hence represses cell cycle progression. Alternatively, AHR may also increase expression of CDKN1A and CDKN1B genes resulting in increased p21 and p27 protein levels which also repress cyclin/CDK activity and maintain the hypo-phosphorylated state of Rb, thereby also reducing cell cycle progression.

This study also further highlights the deregulation in cell cycle of PA and the role the AHR may play in regulating this balance in key cell cycle protein expression. The 31 PA analysed by qPCR exhibited altered CDK–CDKN expression with a general increase in cyclins and CDK gene expression and a reduction in CDKN expression in tumours as compared to normal controls (Fig. 4). Cyclin D1 and cyclin E protein expression were already reported to be increased in a PA when compared to normal controls in other studies (Jordan *et al.* 2000, Turner *et al.* 2000, Saeger *et al.* 2001, Dworakowska *et al*. 2009, Formosa *et al.* 2012). Similarly, CDKN1B protein expression is also found to be significantly reduced in PA (Bamberger *et al.* 1999). In this study, *CDKN1A* (p21) expression has not been found to differ between tumour and control pituitary tissues although non-functional tumours appear to have significantly less p21 protein than GH-secreting tumours. p21 expression has been shown to play a significant role in inducing senescence and in regulating the anti-proliferative role of somatostatin analogues (Chesnokova *et al.* 2008, Monahan *et al.* 2012). The possibility that AHR induces relative changes in the simultaneous or sequential expression of different cell cycle regulatory proteins in order to regulate cell proliferation constitutes a valid hypothesis. According to Marlowe *et al*. (2004) both mechanisms, interaction with Rb and regulation of expression of CDKNs might play an equal and synergistic role in determining the cell cycle repression caused by AHR activity. Figure 6 summarizes how the AHR protein might be acting in pituitary cells, though these two pathways that might be directly or indirectly connected through feedback mechanisms.

This study aimed at identifying novel pathways that might regulate PA behaviour and is the first to identify a mechanistic role for the AHR in pituitary tumourigenesis. Recent studies into the homeostatic roles of this nuclear receptor have elucidated important functions involving cell differentiation, immunity regulation and determination of cell fate in blood cell lineages among others (Singh *et al.* 2009, Vogel *et al.* 2009, Platten *et al.* 2014). Consequently, the AHR is being given increasing attention with regards to its role in normal cell biology above its function as a xenobiotic receptor. This study highlights the complexity of the AHR with its multiple functions in pituitary tissue, from xenobiotic signalling, to endocrine disruption to cell cycle regulation and PA behaviour. Given its close association with AIP, the discovery of AHR as a putative tumour suppressor may shed new light upon the importance of the AIP–AHR interaction in PA susceptibility. These proteins work in a complex network of interactions and cascading effects that are very difficult to elucidate in their entirety. Nonetheless, this study provides irrefutable evidence regarding the putative roles of AHR in the pituitary, which may as yet be revealed to be much more complex. With regards to cell proliferation, the corroboration of results from both *in vivo* specimens and *in vitro* analyses lend weight to the hypothesis that AHR may act as a tumour suppressor in PA.

## Supplementary Material

Supporting Figure 1

Supporting Figure 2

## Declaration of interest

The authors declare that there is no conflict of interest that could be perceived as prejudicing the impartiality of the research reported.

## Funding

R Formosa was funded by the REACH HIGH Scholars Programme – Post-Doctoral Grant. The Grant is part-financed by the European Union, Operational Programme II – Cohesion Policy 2014–2020 *investing in human capital to create more opportunities and promote the wellbeing of society* – European Social Fund. J Vassallo and J Borg were funded by the University of Malta Research Fund Committee Allocation (PHBRP07-02) and Faculty of Medicine and Surgery Funds (MDSIN08-22) and an EMBO Long-Term Fellowship ALTF71-2011.

## Author contribution statement

R Formosa is main investigator and was responsible for the cell culture work, tumour collection, RNA extraction and partial analysis of the microarray and IPA. J Borg carried out the major bioinformatics analysis of the microarrays and IPA. J Vassallo is the project coordinator and provided intellectual and technical assistance.
