# Supplementary figures and images for: Aryl hydrocarbon receptor (AHR) is a potential tumour suppressor in pituitary adenomas
Source: Endocr Relat Cancer. 2017 Jun 22;24(8):445–57. doi: 10.1530/ERC-17-0112 (PMC5541251; doi:10.1530/ERC-17-0112)

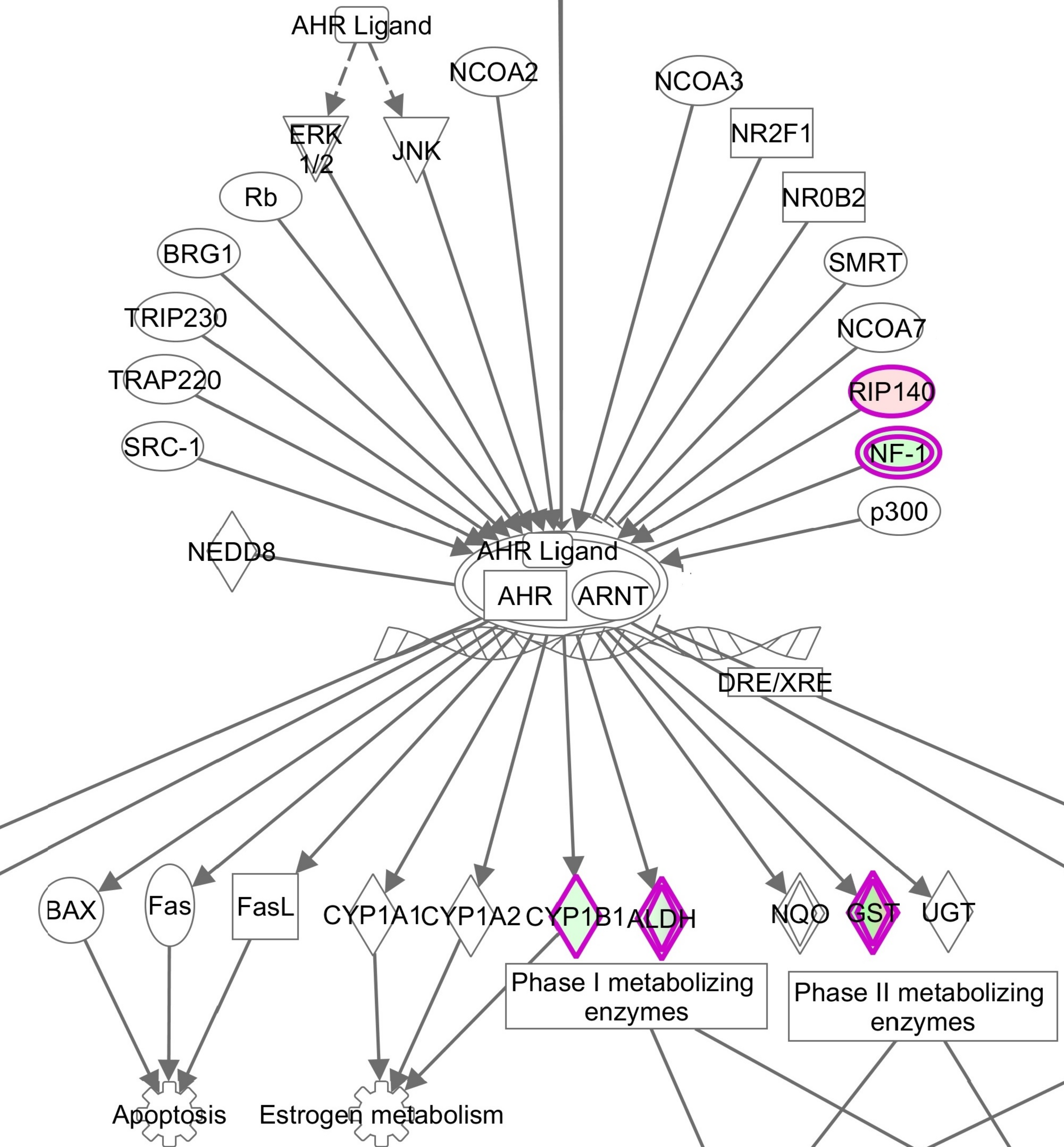

Supplement: Supporting Figure 1 [file erc-24-445-s001.pdf]

**A**

Luciferase/Renilla fold change over untreated cells

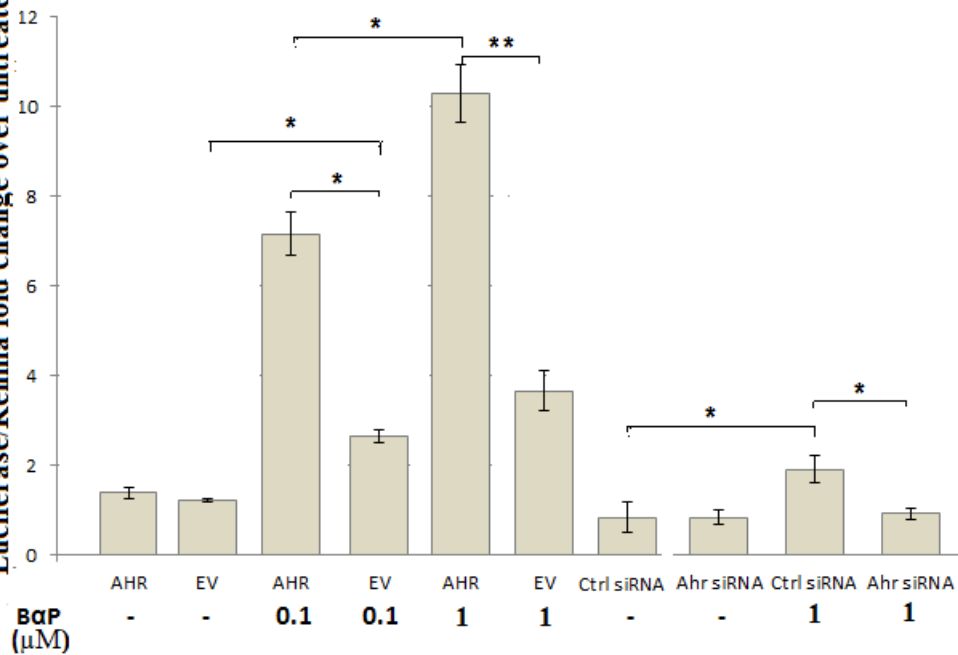**B**

Fold expression change over EV

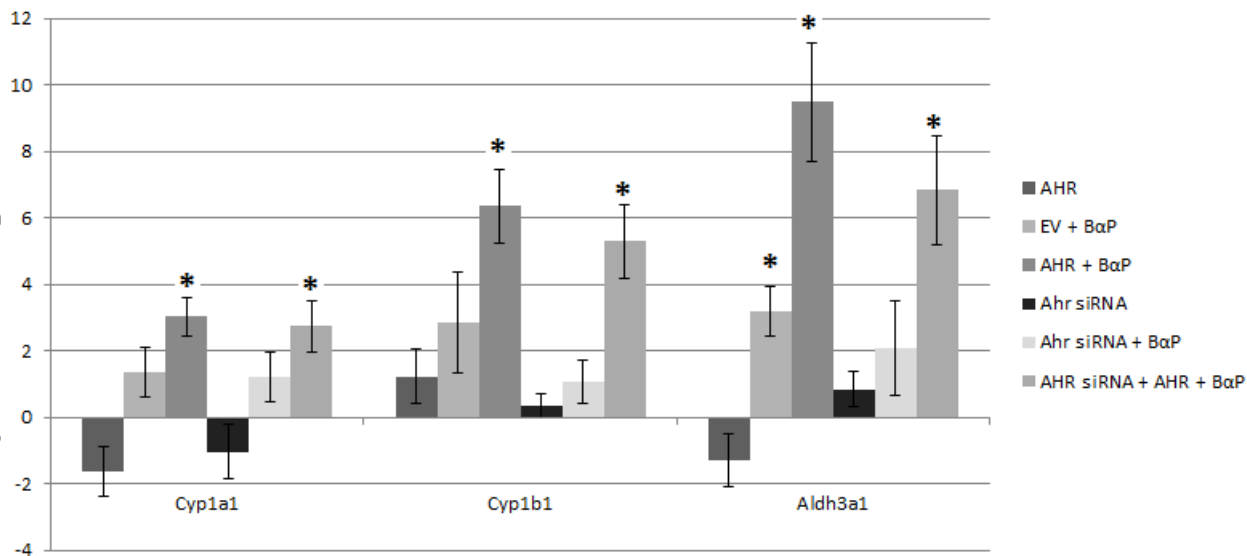

Supplement: Supporting Figure 2 [file erc-24-445-s002.pdf]
